# The Effect of Tanreqing Injection on the Pharmacokinetics of Sirolimus in Rats

**DOI:** 10.1155/2019/1854323

**Published:** 2019-03-10

**Authors:** Feng Zhang, Liang Sun, Jianxiu Zhai, Tianyi Xia, Wei Jiang, Mingming Li, Shouhong Gao, Xia Tao, Wansheng Chen, Yifeng Chai

**Affiliations:** Department of Pharmacy, Changzheng Hospital, Second Military Medical University, Shanghai 200003, China

## Abstract

To evaluate the effect of Tanreqing injection on the pharmacokinetics of sirolimus in rats, a high performance liquid chromatography tandem mass spectrometry method was developed for sirolimus assay in whole blood. Calibration curve of sirolimus was acquired over a concentration ranging from 2.5 to 100 ng/mL with r^2^= 0.9955. The matrix effects and extraction recoveries of sirolimus ranged from 144% to 152% and from 80% to 96%, respectively. The inter- and intraday relative standard deviations were both <10%. The stability investigation showed that the blood samples were stable for 30-day-storage at −20°C, for 8 h storage at room temperature, for 24 h storage in the auto-sampler at 4°C, and for three freeze-thaw cycle process. The pharmacokinetic results demonstrated that the *C*_max_, AUC, and AUMC of sirolimus in rats (7.5 mg/kg, i.g.) were increased after beincoadministration with Tanreqing Injection at 2.5, 5.0, and 7.5 mL/kg (i.v.), respectively, or at 5 min, 2 h, and 4 h (5.0 mL/kg, i.v.) after SRL dosing, respectively. For the first time, the results proved the herb-drug interaction between Tanreqing Injection and sirolimus and accordingly suggested avoiding concurrent reception of those two drugs for patients.

## 1. Introduction

For sirolimus (SRL, rapamycin), a common immunosuppressant, its efficacy came from inhibition on the serine/threonine kinase mammalian target (mTOR) by forming a complex with the immunophilin FK-506-binding protein (FKBP)-12[1]. SRL also exhibits immunosuppressive property by blocking activity of cell cycle progression at the juncture of G1 and S phase and thus suppressing cytokine-mediated T cell proliferation [2]. SRL is metabolized extensively by cytochrome P450 3A (CYP3A) and is a substrate of the drug efflux pump P-glycoprotein. Metabolites contribute to <10% of immunosuppressive activity of the parent compound [3]. As an effective immunosuppressant, SRL has resulted in less nephrotoxicity compared with the calcineurin inhibitors (CNIs), like cyclosporine A (CsA) and tacrolimus (TAC) [4]. However, with narrow therapeutic window (recommended trough concentration range of 5–15 ng/mL), the large intra- and interpatient variability in drug exposure heavily threatened the safety of patients in SRL treatment. What is more, the application of SRL was also limited by its low and variable oral bioavailability (about 14%) [4]. The routine therapeutic drug monitoring (TDM) of SRL blood concentration is essential for patients, to individualize the drug dose and thereby prevent drug toxicity or organ rejection and subsequent drug discontinuation.

Tanreqing Injection (TRQ), a classical TCM formulation, is produced from five herb raw material: Scutellariae Radix, Fel selenarcti, Cornu naemorhedi, Lonicerae japonicae Flos, and Forsythiae fructus. In China [5, 6], it is commonly used to treat acute upper respiratory tract infection and early stage of pneumonia in clinical practice. Our previous chemical profile and metabolism profile analyses have revealed that TRQ contains flavones from Scutellariae Radix and Forsythiae fructus, cholic acids from Fel selenarcti, amino acids from Cornu naemorhedi, and phenolic acids from Lonicerae japonicae Flos as its major constituents, as well as the latest reports in other labtory [7–9].

Lately, the safety of SRL application was concerned in combined drug administration treatment, in which case drug/herb-drug interactions (DDI/HDI) may result in some adverse effects (AEs). For example, an article described that a drug-drug interaction between azole antifungals or macrolide antibiotics and SRL in five patients led to an increase of SRL exposure [10], while another reported a decrease of SRL exposure in the presence of rifampin and phenytoin concomitantly[11]. In 2011, a sudden rise of SRL level (23.5 ng/mL, 100%) was found in a renal transplantation patient in our hospital, who was treated with SRL (2 mg/day for 10 years) and Cellcept (mycophenolate mofetil, 750 mg twice a day) and then was given TRQ (30 mL/day for two days) for pneumonia. Under excessive exposure of SRL, the patient suffered from thrombocytopenia and leukopenia due to high trough concentration of SRL. No pharmacokinetic DDI was recognized for Cellcept and SRL [12]. Afterwards, with withdrawal of TRQ and constant prescription of SRL and Cellcept, the trough SRL concentration was decreased to 15.4 ng/mL, and the previous side effects for the patient disappeared accordingly. This finding not only emphasized the need for a close TDM of SRL concentrations in transplant patients but also indicated a suspicious HDI between SRL and TRQ.

Immunoassay was reported as a reliable and convenient method for SRL blood concentration detection. However, it was also known that immunoassay might lead to overestimation of SRL concentration as a result of cross-reactivity with metabolites [13]. Therefore, several high performance liquid chromatography coupled to tandem mass spectrometry (LC-MS/MS) methods have been developed for the quantitation of SRL or multiple immunosuppressants concentrations in whole blood, with improved specificity and sensitivity [14–18]

As above mentioned, no literatures were reported about the herb-drug interactions between SRL and TRQ. In order to investigate the potential HDI between SRL and TRQ, a LC-MS/MS method was established to evaluate the effect on the pharmacokinetics of SRL in rats after its co-administration with TRQ (1) on three different doses and (2) at three different times.

## 2. Materials and Methods

### 2.1. Chemicals and Reagents

**Figure 1:**
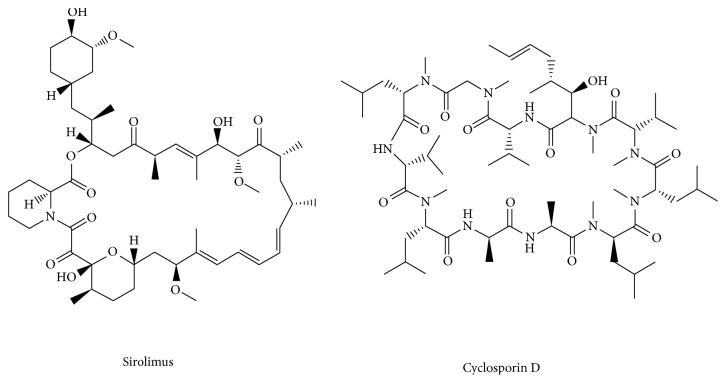
Chemical structures of sirolimus and cyclosporin D.

SRL and cyclosporin D (internal standard, IS) (Figure 1) with analytical reference standards (purity ≥ 99.0%) were purchased from Sigma-Aldrich Co. (St. Louis, MO, USA). SRL tablets (1 mg per tablet) were obtained from Wyeth Company (USA). TRQ injections (10 mL per vial) were provided from Shanghai Kaibao Pharmaceutical Co., Ltd (China). MS-grade acetonitrile and methanol were purchased from Merck (Darmstadt, Germany). Deionized water was prepared using the Milli-Q system (Millipore, Bedford, MA, USA) and was used for all preparations. Other reagents were of analytical grade.

Stock solutions (1 mg/mL) of SRL and IS were prepared in methanol, respectively. SRL stock solution was diluted in methanol-water (50:50) to obtain a series of working solutions, at concentrations of 25, 50, 100, 200, 400, 500, 800, and 1000 ng/mL. Calibration standards were prepared by dilutions of the above working solutions with appropriate blank rat blood to attain concentrations of 2.5, 5, 10, 20, 40, 50, 80, and 100 ng/mL. QC samples were obtained with concentrations of 5, 40, and 80 ng/mL. All solutions were stored at −20°C.

### 2.2. LC-MS/MS Condition

Analysis was carried out on an Agilent 1290 series HPLC coupled with an Agilent 6460 triple-quadrupole mass spectrometer that was equipped with jet stream electrospray ionization (ESI). Data acquisition and processing was performed on Agilent 6460 Quantitative Analysis version B.01.02 analyst data processing software (Agilent Corporation, Santa Clara, CA, USA). A Poroshell 120 SB-C18 (2.7 *μ*m, 2.1 mm × 75 mm, I.D. Agilent, USA) was kept at 55°C and a flow rate of 0.5 mL/min. Mobile phases consisted of methanol with 0.2% formic acid and 10 mM ammonium acetate (A) and 0.2% aqueous formic acid solution with 10 mM ammonium acetate (B). Gradient elution program was used for analysis: 0–3 min, 60% A; 3–5 min, 60–95% A; 5–8 min, 95%A. Injection volume was set at 10 *μ*L.

Nitrogen (purity 99.9999%) was applied as the collision gas (0.1 MPa). The detector was operated in the positive mode. The conditions of the source parameters were: capillary voltage 4.0 kV, nebulizer 35 psi, drying gas temperature 225°C, drying gas flow 10 L/min, sheath gas temperature 325°C and sheath gas flow 12 L/min. The optimized multiple reaction monitoring (MRM) conditions of the analytes were* m/z *931.6*⟶*864.5 and fragmentation/collision energy 170 V/13 eV for SRL and* m/z *1233.9*⟶*1216.9 and fragmentation/collision energy 190 V/17 eV for IS.

### 2.3. Sample Preparation

100 *μ*L blood was added with 200 *μ*L 0.4 M zinc sulfate aqueous solution: methanol precipitating solution (1:4, v/v, containing IS 200 ng/mL), followed by vortex-mixing (2 min) and centrifugation (12000 rpm, 10 min). The sample supernatant (200 *μ*L) was obtained and then loaded to LC-MS/MS system for analysis.

### 2.4. Method Validation

Method validation was performed according to US FDA guidance [19]. Selectivity of the analyte was evaluated by comparing the chromatograms of six different blank rat blood, with those of blood spiked with SRL and IS, as well as those of the rat blood sample after drug administration. Calibration curves were obtained by plotting the peak-area ratio between SRL and IS against the nominal concentrations. Linearity was evaluated by weighted (1/*x*^2^) least squares linear regression analysis. The lower limit of quantification (LLOQ) was defined as the lowest SRL concentration with a signal-to-noise ratio of 10:1 and evaluated by analyzing spiked blood samples prepared in six replicates. The intra- and interday precisions and accuracies were obtained by analyzing five replicates of QC samples in three levels. The precision was defined as the relative standard deviation (RSD) and accuracy was expressed as relative error (RE). Matrix effect was determined by comparing the peak areas of SRL and IS spiked in extracted QC samples with those of analytes in standard solutions at equivalent concentrations. Extraction recovery was evaluated by comparing the peak areas of SRL and IS spiked in extracted QC samples with those of un-extracted standard solutions containing the equivalent amount of SRL. Stability evaluation was performed in the following conditions. Long-term stability was tested for the samples that were kept at −20°C for 30 days before analysis. Short-term stability was tested for the samples that were kept at room temperature for 8 h before analysis. Post-preparative stability was tested when the samples were kept in an auto-sampler at 4°C for 24 h before analysis. Freeze and thaw stability was tested for three freeze-thaw cycles when the samples were stored at −20°C for 24 h and thawed at room temperature.

### 2.5. Application of the Assay and HDI Investigation of SRL and TRQ

All animal protocols were approved by Animal Ethics Committee of Second Military Medical University. Sprague-Dawley rats (adult male, 200 ± 20 g) were obtained from Shanghai SLAC laboratory animal Co. Ltd. (Shanghai, China). Rats were quarantined for one week prior to study, and were housed in well ventilated cages at 20 ± 1°C and 50 ± 10% air humidity while on a 12-h light-dark cycle. All rats were fasted with free access to water for 12 h before experiment.

In order to investigate the effect of different dose levels and dosing time of TRQ on the pharmacokinetics of SRL analysis, SD rats were divided into 6 groups of 6 animals each: control group-SRL (7.5 mg/kg, i.g.) alone, group A1-coadministration of SRL (7.5 mg/kg, i.g.) with TRQ (2.5 mL/kg, i.v.), group A2-co-administration of SRL (7.5 mg/kg, i.g.) with TRQ (5 mL/kg, i.v.), and group A3-co-administration of SRL (7.5 mg/kg, i.g.) with TRQ (10 mL/kg, i.v.). TRQ were given to all rats at 5 min after SRL dosing in groups A1–A3.

In order to investigate the effect of different dosing time of TRQ on the pharmacokinetics of SRL analysis, SD rats were divided into 4 groups of 6 animals each: control group-SRL (7.5 mg/kg, i.g.) alone, and the rest rats in groups B1–B3 were treated with SRL (7.5 mg/kg, i.g.) and TRQ (5 mL/kg, i.v.). TRQ were given to rats at 5 min, 2 h and 4 h after SRL dosing in groups B1–B3, respectively. See the experimental protocol in supplementary Figure S1.

Blood samples (0.5 mL) were collected into heparinized tubes via retro-orbital plexus at pre-dose (0), 20, 40 min, 1, 2, 4, 6, 8, 12, 24, 36, and 48 h post-dose. Blood samples were stored at −20°C prior to analysis. Pharmacokinetic parameters (including *C*_max_, *T*_max_, *t*_1/2_,* AUC*,* AUMC*, and* MRT*) were calculated on Drug and Statistics DAS 3.2.6 (Mathematical Pharmacology Professional Committee of China, Shanghai, China), using noncompartmental pharmacokinetic analysis. The differences between groups were evaluated by Student's t test and were considered to be significant at ^*∗*^P<0.05 and ^*∗∗*^P<0.01.

## 3. Results and Discussion

### 3.1. Method Development

Cyclosporine D was served as IS as the previous report [15]. As MRM mode in LC-MS/MS detection allowed simultaneous quantitative determination of drugs with high specificity, low detection limits, and short time of analysis [19], it was applied to the analysis of SRL and IS. In order to release SRL from the erythrocytes where SRL was predominantly distributed (about 95%), zinc sulphate was used to lyse the erythrocytes. It was thought that the further sample treatment like solid-phase extraction or liquid-liquid extraction would contribute to eliminate the phospholipids from blood, and then increase the efficiency of SRL recovery [18, 19]. However, that step would be time- and cost-consuming, hard to be applied in the high-throughput TDM. Therefore, a one-step PPT protocol, in conjunction with a proper LC method, was optimized to eliminate the phospholipids in the initial 3 min. The addition of 0.2% formic acid and 10 mM ammonium acetate in phases A and B was favorable to enhance the MS response for both SRL and IS. After checking different gradients in mobile phases and other conditions, the best results were obtained with the conditions described in “**LC-MS/MS condition**”.

### 3.2. Method Validation

Calibration curve of SRL (y=0.0024x -0.0026) exhibited effective linearity (R^2^= 0.9955) over a range of 2.5–100 ng/mL, with LLOQ of 2.5 ng/mL. In detail, inter- and intraday precisions and accuracies for SRL had RSD values less than 10%. The extraction recovery was between 80% and 96%. The matrix effect of SRL was between 144% and 152%. Stability investigation showed that the RE values for SRL were less than 15% in rat blood for 30-day-storage at −20°C, below 8% for 8-h-storage at room temperature, less than 10% for 24-h-storage in the auto-sampler at 4°C, and below 20% for three freeze-thaw cycle process, which indicated that the blood samples were stable during the entire experiment. All the above results were within the ranges requested by the FDA for bioanalytical method validation and could be applied to the SRL pharmacokinetic study in rat.

### 3.3. Results of Pharmacokinetic Study and HDI Investigation

**Figure 2:**
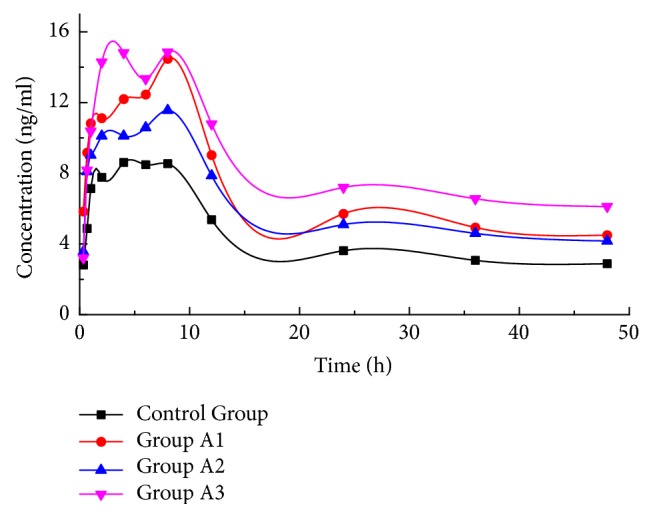
Mean blood concentration-time profiles of sirolimus after the administration of TRQ at different doses.

To evaluate the effect of different dose levels of TRQ on the pharmacokinetics of SRL, SRL pharmacokinetic data of control group and group A1–A3 were demonstrated in Table 1. The corresponding mean blood concentration-time profiles were showed in Figure 2. Several pharmacokinetic parameters presented an obvious increase trend in groups A1–A3 when compared with those in control group, and Cmax, AUC0→t and AUMC showed significant difference. Compared with that of control group (9.487±1.479 ng/ml), the *C*_max_ of A1, A2, and A3 groups were significantly increased by 64% (15.546±4.234 ng/ml, p < 0.01), 28% (12.201±1.153 ng/ml, p < 0.01), and 70% (16.043±1.437 ng/ml, p < 0.01), respectively. In parallel, the *AUC*_0→t_ of A1, A2, and A3 groups were significantly increased by 59% (349.346±69.514 ng·h/mL, p < 0.01), 39% (305.875±31.417 ng·h/mL, p < 0.01), and 92% (421.518±42.166 ng·h/mL, p < 0.01), respectively, when compared to control group (219.673±32.306 ng·h/mL). As for* AUMC*, it was significantly increased by 60% (6577.191±1205.275 ng·h/mL, p < 0.01), 44% (5935.337±819.676 ng·h/mL, p < 0.01), and 104% (8394.303±820.509 ng·h/mL, p < 0.01), respectively, when compared to control group (4122.178±593.331 ng·h/mL). It revealed that coadministration of SRL with single-dose TRQ could markedly increase the blood concentration of SRL. In the lowest dose of TRQ group (2.5 mL/kg), the three parameters of SRL mentioned above were increased by approximately 1-fold, suggesting that the HDI might appear even with lowest dose of TRQ coadministration. However, no change in *T*_max_ was observed with or without TRQ coadministration. Also, values of *t*_1/2_ and* MRT* were similar with or without TRQ co-administration. The above observations suggested a potential HDI between SRL and TRQ.

**Figure 3:**
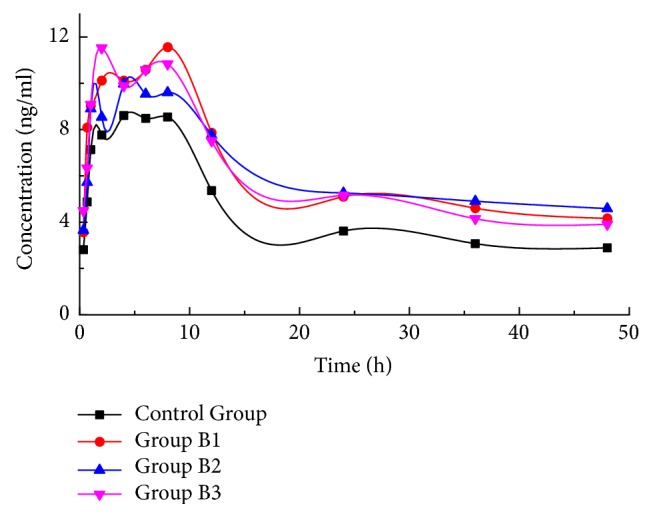
Mean blood concentration-time profiles of sirolimus after the administration of TRQ at different time.

To evaluate the effect of different dosing time of TRQ on the pharmacokinetics of SRL, SRL pharmacokinetic data of control group and groups B1–B3 were listed in Table 2. The relevant mean blood concentration-time profiles were shown in Figure 3. Among those parameters, only *AUC*_0→t_ showed significant difference (P< 0.01) in groups B1–B3 when compared with those in control group, and *AUC*_0→t_ in group B3 was smaller than that in groups B1 and B2. *T*_max_ and* AUMC* presented in a similar trend with *AUC*_0→t_ (without significance). In detail, compared with that of control group (219.673±32.306 ng·h/mL), the *AUC*_0→t_ of B1, B2, and B3 groups were significantly increased by 39% (305.875±31.417 ng·h/mL, p < 0.01), 37% (301.677±73.315 ng·h/mL, p < 0.01), and 35% (296.211±65.286 ng·h/mL, p < 0.01), respectively. As for* AUMC*, it was significantly increased by 44% (5935.337±819.676 ng·h/mL, p < 0.01), 49% (6157.161±1329.936 ng·h/mL, p < 0.01), and 37% (5635.173±1350.868 ng·h/mL, p < 0.01), respectively, when compared to control group (4122.178±593.331 ng·h/mL). Therefore, it could be concluded that the TRQ significantly enhanced the* AUC* of SRL when TRQ was administrated at the same time with SRL or 2 hours after SRL administration. This effect was not obvious when TRQ was administrated 4 hours after SRL administration. Besides, the pharmacokinetic profiles of SRL also showed the similar curves in the groups B1–B3. Based on the effect of different dose levels and different dosing time of TRQ, it could be found that the* AUC*s in groups A1-A3 and groups B1–B3 were higher than those in control groups, which indicated that TRQ might cause overexposure of SRL in patients receiving SRL along with TRQ.

SRL is a substrate for CYP3A and P-gp. It is not sure that the five TCMs from TRQ or its main components inhibit the hepatic and intestinal CYP3A/P-gp system. However, it have been reported by some literatures that baicalin, a main component from Scutellariae Radix in TRQ, could inhibit hepatic CYP3A activity as well as P-glycoprotein efflux pump in the small intestine [20–22]. Therefore, the affected SRL pharmacokinetics by TRQ might be mainly due to the inhibition of CYP3A-mediated metabolism and P-gp-mediated transport by baicalin. The complex mixture of chemical constituents in herbal medicines or TCMs, which are usually believed to be medicinally efficacious, are yet to be fully characterized [23]. There might be other compounds responsible for the HDI mechanism. In future, further studies will be needed to clarify this point.

Actually, like the pharmacokinetic profile of the mean value of SRL in the stable renal transplant patients [24], the control group of SRL did not show the obvious double peaks, which were only found in the SRL-TRQ combination treatment groups. Because of the extensive distribution in red blood cells of SRL (94.5%) [24], zinc sulfate was applied as the erythrolysis agent to release SRL in the circulating red blood cells. In this study, the absorption of SRL fluctuated. The reason could be that the absorption rate of SRL varied in different parts of the intestine in rats [25]. Thus, the double peaks for SRL in the TRQ pretreatment groups might be attributed to the effect of chemical compounds from TRQ on the elimination of SRL, which should be investigated in our future study.

It was reported that SRL pharmacokinetics was associated with CYP3A and MDR1 genetic polymorphisms, but we failed to determine the relevant genetic information of the patient in the introduction. If the patient expressed the CYP3A5 enzyme (CYP3A5*∗*1 carriers), he might need more SRL to reach target concentrations when compared with those with CYP3A5*∗*3/*∗*3 carriers [26]. It might lead to a risk of drug toxicity, especially when a potential HDI existed.

## 4. Conclusions

A simple and accurate LC-MS/MS method was developed and validated for SRL assay in rat blood following a quick PPT procedure, which demonstrated good selectivity, linearity, precision and accuracy, matrix effect and recovery, and stability. This assay was successfully applied to evaluate the pharmacokinetics of SRL, when it was administrated alone and coadministrated with TRQ in rat. The results showed that different dose levels and different dosing time of TRQ would increase SRL blood concentration and exposure, indicating a potential HDI between TRQ and SRL. The study provided valuable information for SRL treatment: from the pharmacokinetic point of view, coadministration with TRQ or baicalin-derived products was not recommended in clinic. A systematical TDM for SRL was greatly encouraged for safety and would help to discover a possible DDI/HDI.

## Figures and Tables

**Table 1:** Pharmacokinetic parameters of SRL after the administration of TRQ at different doses.

Parameter	Control Group	Group A1	Group A2	Group A3
*T*max (h)	4.167±2.563	6.500±2.811	5.167±3.251	6.333±2.658
*C*max (ng/mL)	9.487±1.479	15.546±4.234^*∗∗*^	12.201±1.153^*∗∗*^	16.043±1.437^*∗∗*^
*t*_1/2 _ (h)	68.144±63.013	57.543±46.635	47.119±43.595	118.158±186.146
AUC_0→t_ (ng·h/mL)	219.673±32.306	349.346±69.514^*∗∗*^	305.875±31.417^*∗∗*^	421.518±42.166^*∗∗*^
AUC_0→*∞*_ (ng·h/mL)	493.458±271.184	684.035±233.463	588.668±339.317	1456.282±1708.762
AUMC_0→t_ (ng·h/mL)	4122.178±593.331	6577.191±1205.275^*∗∗*^	5935.337±819.676^*∗∗*^	8394.303±820.509^*∗∗*^
MRT_0→t_ (h)	18.772±0.456	18.881±0.946	19.343±0.976	19.919±0.275^*∗∗*^

^*∗∗*^P<0.01, compared with control group.

**Table 2:** Pharmacokinetic parameters of SRL after the administration of TRQ at different time.

Parameter	Control Group	Group B1	Group B2	Group B3
*T*max (h)	4.167±2.563	5.167±3.251	5.001±4.472	3.001±2.449
*C*max (ng/mL)	9.487±1.479	12.201±1.153^*∗∗*^	11.643±3.648	12.699±3.479
*t*_1/2_ (h)	68.144±63.013	47.119±43.595	65.408±27.871	33.299±14.359
AUC_0→t_ (ng·h/mL)	219.673±32.306	305.875±31.417^*∗∗*^	301.677±73.315^*∗∗*^	296.211±65.286^*∗∗*^
AUC_0→*∞*_ (ng·h/mL)	493.458±271.184	588.668±339.317	694.003±91.568	456.371±133.544
AUMC_0→t_ (ng·h^2^/mL)	4122.178±593.331	5935.337±819.676^*∗∗*^	6157.161±1329.936^*∗∗*^	5635.173±1350.868^*∗∗*^
MRT_0→t_ (h)	18.772±0.456	19.343±0.976	20.527±0.801^*∗∗*^	18.962±0.716

^*∗∗*^P<0.01, compared with control group.

## Data Availability

The data used to support the findings of this study are available from the corresponding author upon request.

## Supplementary Materials

Supplementary Materials Please fine the experimental protocol in supplementary Figure S1.
