# Supplementary material for: The Effect of Tanreqing Injection on the Pharmacokinetics of Sirolimus in Rats
Source: Biomed Res Int. 2019 Mar 10;2019:1854323. doi: 10.1155/2019/1854323 (PMC6431440; doi:10.1155/2019/1854323)

Supplementary materials

The effect of Tanreqing injection on the pharmacokinetics of sirolimus in rats

Fig S1. the experimental protocol

HDI investigation groups

Dose investigation:

A1: SRL (7.5 mg/kg, i.g.) + TRQ (2.5 mL/kg, i.v.)
*A2: SRL (7.5 mg/kg, i.g.) + TRQ (5 mL/kg, i.v.)
A3: SRL (7.5 mg/kg, i.g.) + TRQ (10 mL/kg, i.v.)

Time-interval investigation:

Control group: SRL (7.5 mg/kg, i.g.) alone
*B1: SRL (7.5 mg/kg, i.g.) + TRQ (5 mL/kg, i.v.)--time interval: 5 min
B2: SRL (7.5 mg/kg, i.g.) + TRQ (5 mL/kg, i.v.)--time interval: 2 h
B3: SRL (7.5 mg/kg, i.g.) + TRQ (5 mL/kg, i.v.)--time interval: 4 h

*A2 and B1 are the same group.

Graphic abstract

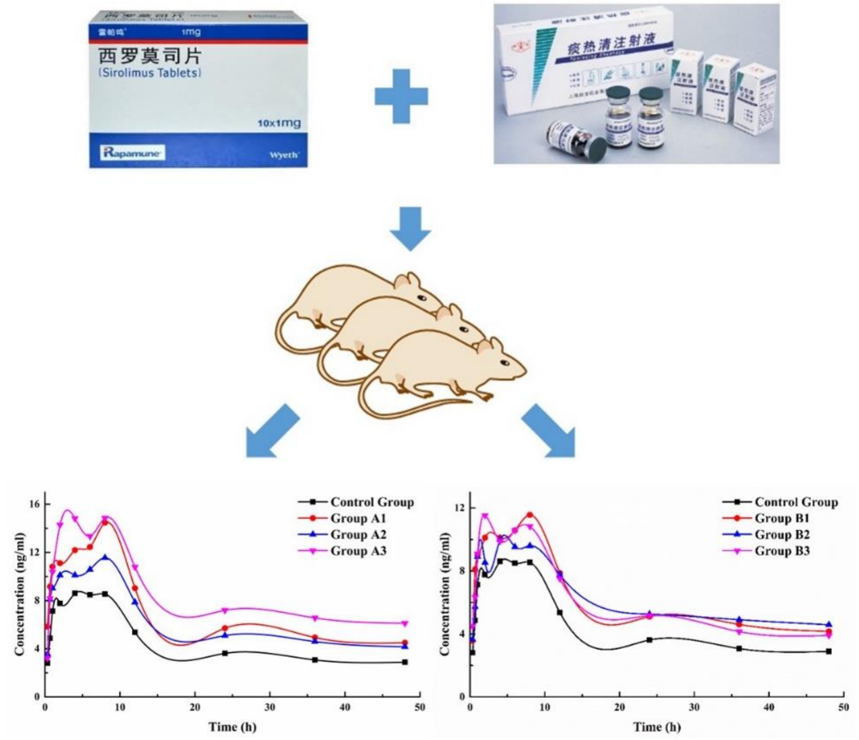

Supplement: Supplementary Materials — Please fine the experimental protocol in supplementary Figure S1. [file 1854323.f1.pdf]
